# Supplementary material for: Time-series transcriptome analysis identified differentially expressed genes in broiler chicken infected with mixed Eimeria species
Source: Front Genet. 2022 Aug 8;13:886781. doi: 10.3389/fgene.2022.886781 (PMC9393255; doi:10.3389/fgene.2022.886781)
Supplement: Supplementary file 2 [file DataSheet1.ZIP › 4dpi_KEGG.Gsea.1625100298584/gsea_report_for_1_1625100298584.html]

Report for 1 1625100298584 [GSEA]

| GS  follow link to MSigDB | GS DETAILS | SIZE | ES | NES | NOM p-val | FDR q-val | FWER p-val | RANK AT MAX | LEADING EDGE || 1 | KEGG\_STEROID\_BIOSYNTHESIS | Details ... | 12 | 0.97 | 2.39 | 0.000 | 0.000 | 0.000 | 418 | tags=100%, list=3%, signal=104% |
| 2 | KEGG\_ADIPOCYTOKINE\_SIGNALING\_PATHWAY | Details ... | 52 | 0.53 | 1.86 | 0.001 | 0.050 | 0.094 | 2881 | tags=52%, list=24%, signal=68% |
| 3 | KEGG\_TERPENOID\_BACKBONE\_BIOSYNTHESIS | Details ... | 12 | 0.75 | 1.86 | 0.002 | 0.034 | 0.094 | 299 | tags=33%, list=2%, signal=34% |
| 4 | KEGG\_RIG\_I\_LIKE\_RECEPTOR\_SIGNALING\_PATHWAY | Details ... | 39 | 0.55 | 1.83 | 0.002 | 0.036 | 0.133 | 982 | tags=21%, list=8%, signal=22% |
| 5 | KEGG\_CYTOSOLIC\_DNA\_SENSING\_PATHWAY | Details ... | 25 | 0.58 | 1.74 | 0.007 | 0.083 | 0.329 | 505 | tags=24%, list=4%, signal=25% |
| 6 | KEGG\_PPAR\_SIGNALING\_PATHWAY | Details ... | 52 | 0.47 | 1.64 | 0.007 | 0.183 | 0.654 | 1519 | tags=42%, list=13%, signal=48% |
| 7 | KEGG\_TIGHT\_JUNCTION | Details ... | 89 | 0.41 | 1.58 | 0.004 | 0.263 | 0.835 | 2614 | tags=36%, list=22%, signal=46% |
| 8 | KEGG\_HISTIDINE\_METABOLISM | Details ... | 22 | 0.54 | 1.56 | 0.026 | 0.273 | 0.883 | 1072 | tags=32%, list=9%, signal=35% |
| 9 | KEGG\_APOPTOSIS | Details ... | 63 | 0.43 | 1.55 | 0.012 | 0.272 | 0.910 | 1333 | tags=22%, list=11%, signal=25% |
| 10 | KEGG\_GLYCOSPHINGOLIPID\_BIOSYNTHESIS\_LACTO\_AND\_NEOLACTO\_SERIES | Details ... | 17 | 0.56 | 1.53 | 0.045 | 0.289 | 0.942 | 331 | tags=18%, list=3%, signal=18% |
| 11 | KEGG\_HOMOLOGOUS\_RECOMBINATION | Details ... | 23 | 0.53 | 1.52 | 0.044 | 0.276 | 0.951 | 1046 | tags=30%, list=9%, signal=33% |
| 12 | KEGG\_GNRH\_SIGNALING\_PATHWAY | Details ... | 66 | 0.41 | 1.52 | 0.018 | 0.267 | 0.959 | 2262 | tags=30%, list=19%, signal=37% |
| 13 | KEGG\_TAURINE\_AND\_HYPOTAURINE\_METABOLISM | Details ... | 5 | 0.77 | 1.50 | 0.047 | 0.287 | 0.977 | 661 | tags=40%, list=6%, signal=42% |
| 14 | KEGG\_JAK\_STAT\_SIGNALING\_PATHWAY | Details ... | 90 | 0.38 | 1.46 | 0.016 | 0.348 | 0.993 | 2691 | tags=34%, list=22%, signal=44% |
| 15 | KEGG\_ADHERENS\_JUNCTION | Details ... | 63 | 0.40 | 1.45 | 0.035 | 0.346 | 0.996 | 2609 | tags=37%, list=22%, signal=46% |
| 16 | KEGG\_TOLL\_LIKE\_RECEPTOR\_SIGNALING\_PATHWAY | Details ... | 67 | 0.39 | 1.45 | 0.031 | 0.331 | 0.996 | 1229 | tags=19%, list=10%, signal=21% |
| 17 | KEGG\_DNA\_REPLICATION | Details ... | 29 | 0.45 | 1.38 | 0.081 | 0.480 | 1.000 | 3366 | tags=59%, list=28%, signal=81% |
| 18 | KEGG\_VASCULAR\_SMOOTH\_MUSCLE\_CONTRACTION | Details ... | 75 | 0.37 | 1.38 | 0.047 | 0.466 | 1.000 | 3608 | tags=41%, list=30%, signal=59% |
| 19 | KEGG\_GLYCOSPHINGOLIPID\_BIOSYNTHESIS\_GLOBO\_SERIES | Details ... | 12 | 0.55 | 1.35 | 0.131 | 0.515 | 1.000 | 110 | tags=17%, list=1%, signal=17% |
| 20 | KEGG\_FOLATE\_BIOSYNTHESIS | Details ... | 9 | 0.57 | 1.32 | 0.160 | 0.569 | 1.000 | 3739 | tags=67%, list=31%, signal=97% |
| 21 | KEGG\_LONG\_TERM\_DEPRESSION |  | 42 | 0.40 | 1.32 | 0.101 | 0.547 | 1.000 | 1663 | tags=21%, list=14%, signal=25% |
| 22 | KEGG\_SPHINGOLIPID\_METABOLISM |  | 31 | 0.42 | 1.31 | 0.123 | 0.558 | 1.000 | 1889 | tags=32%, list=16%, signal=38% |
| 23 | KEGG\_P53\_SIGNALING\_PATHWAY |  | 55 | 0.37 | 1.31 | 0.100 | 0.540 | 1.000 | 2264 | tags=31%, list=19%, signal=38% |
| 24 | KEGG\_TGF\_BETA\_SIGNALING\_PATHWAY |  | 62 | 0.36 | 1.30 | 0.089 | 0.531 | 1.000 | 3917 | tags=50%, list=33%, signal=74% |
| 25 | KEGG\_ENDOCYTOSIS |  | 141 | 0.31 | 1.30 | 0.053 | 0.533 | 1.000 | 3835 | tags=45%, list=32%, signal=66% |
| 26 | KEGG\_TYPE\_I\_DIABETES\_MELLITUS |  | 11 | 0.53 | 1.29 | 0.174 | 0.529 | 1.000 | 1947 | tags=45%, list=16%, signal=54% |
| 27 | KEGG\_SYSTEMIC\_LUPUS\_ERYTHEMATOSUS |  | 22 | 0.44 | 1.28 | 0.153 | 0.534 | 1.000 | 1109 | tags=27%, list=9%, signal=30% |
| 28 | KEGG\_CELL\_CYCLE |  | 103 | 0.32 | 1.27 | 0.099 | 0.559 | 1.000 | 3700 | tags=43%, list=31%, signal=61% |
| 29 | KEGG\_OLFACTORY\_TRANSDUCTION |  | 14 | 0.48 | 1.24 | 0.205 | 0.626 | 1.000 | 2262 | tags=36%, list=19%, signal=44% |
| 30 | KEGG\_GLYCEROLIPID\_METABOLISM |  | 36 | 0.37 | 1.22 | 0.191 | 0.666 | 1.000 | 1941 | tags=39%, list=16%, signal=46% |
| 31 | KEGG\_SPLICEOSOME |  | 96 | 0.31 | 1.21 | 0.146 | 0.674 | 1.000 | 4119 | tags=42%, list=34%, signal=63% |
| 32 | KEGG\_FC\_GAMMA\_R\_MEDIATED\_PHAGOCYTOSIS |  | 74 | 0.32 | 1.20 | 0.165 | 0.698 | 1.000 | 1986 | tags=28%, list=17%, signal=34% |
| 33 | KEGG\_CYTOKINE\_CYTOKINE\_RECEPTOR\_INTERACTION |  | 134 | 0.29 | 1.18 | 0.154 | 0.715 | 1.000 | 2475 | tags=29%, list=21%, signal=36% |
| 34 | KEGG\_NOD\_LIKE\_RECEPTOR\_SIGNALING\_PATHWAY |  | 37 | 0.36 | 1.18 | 0.212 | 0.696 | 1.000 | 2138 | tags=27%, list=18%, signal=33% |
| 35 | KEGG\_NATURAL\_KILLER\_CELL\_MEDIATED\_CYTOTOXICITY |  | 67 | 0.32 | 1.18 | 0.195 | 0.692 | 1.000 | 1397 | tags=19%, list=12%, signal=22% |
| 36 | KEGG\_AMYOTROPHIC\_LATERAL\_SCLEROSIS\_ALS |  | 36 | 0.36 | 1.17 | 0.228 | 0.690 | 1.000 | 2465 | tags=31%, list=21%, signal=38% |
| 37 | KEGG\_PATHOGENIC\_ESCHERICHIA\_COLI\_INFECTION |  | 44 | 0.35 | 1.17 | 0.229 | 0.681 | 1.000 | 3867 | tags=48%, list=32%, signal=70% |
| 38 | KEGG\_ALDOSTERONE\_REGULATED\_SODIUM\_REABSORPTION |  | 28 | 0.38 | 1.17 | 0.246 | 0.669 | 1.000 | 3009 | tags=43%, list=25%, signal=57% |
| 39 | KEGG\_ETHER\_LIPID\_METABOLISM |  | 20 | 0.41 | 1.17 | 0.255 | 0.657 | 1.000 | 1176 | tags=25%, list=10%, signal=28% |
| 40 | KEGG\_REGULATION\_OF\_ACTIN\_CYTOSKELETON |  | 155 | 0.27 | 1.14 | 0.193 | 0.713 | 1.000 | 2759 | tags=32%, list=23%, signal=41% |
| 41 | KEGG\_ABC\_TRANSPORTERS |  | 33 | 0.35 | 1.13 | 0.275 | 0.729 | 1.000 | 792 | tags=15%, list=7%, signal=16% |
| 42 | KEGG\_PANCREATIC\_CANCER |  | 57 | 0.32 | 1.13 | 0.260 | 0.718 | 1.000 | 3562 | tags=40%, list=30%, signal=57% |
| 43 | KEGG\_ALPHA\_LINOLENIC\_ACID\_METABOLISM |  | 9 | 0.49 | 1.12 | 0.329 | 0.730 | 1.000 | 448 | tags=22%, list=4%, signal=23% |
| 44 | KEGG\_FOCAL\_ADHESION |  | 158 | 0.27 | 1.11 | 0.242 | 0.738 | 1.000 | 2885 | tags=32%, list=24%, signal=42% |
| 45 | KEGG\_GRAFT\_VERSUS\_HOST\_DISEASE |  | 6 | 0.54 | 1.11 | 0.363 | 0.722 | 1.000 | 769 | tags=33%, list=6%, signal=36% |
| 46 | KEGG\_MELANOMA |  | 48 | 0.32 | 1.11 | 0.286 | 0.713 | 1.000 | 2988 | tags=35%, list=25%, signal=47% |
| 47 | KEGG\_ECM\_RECEPTOR\_INTERACTION |  | 64 | 0.31 | 1.11 | 0.283 | 0.700 | 1.000 | 3082 | tags=42%, list=26%, signal=56% |
| 48 | KEGG\_AUTOIMMUNE\_THYROID\_DISEASE |  | 8 | 0.50 | 1.10 | 0.367 | 0.701 | 1.000 | 769 | tags=25%, list=6%, signal=27% |
| 49 | KEGG\_NICOTINATE\_AND\_NICOTINAMIDE\_METABOLISM |  | 18 | 0.40 | 1.10 | 0.331 | 0.694 | 1.000 | 2246 | tags=39%, list=19%, signal=48% |
| 50 | KEGG\_ALLOGRAFT\_REJECTION |  | 7 | 0.52 | 1.10 | 0.374 | 0.686 | 1.000 | 769 | tags=29%, list=6%, signal=31% |
| 51 | KEGG\_PROXIMAL\_TUBULE\_BICARBONATE\_RECLAMATION |  | 16 | 0.41 | 1.09 | 0.352 | 0.685 | 1.000 | 725 | tags=25%, list=6%, signal=27% |
| 52 | KEGG\_MISMATCH\_REPAIR |  | 20 | 0.38 | 1.09 | 0.340 | 0.672 | 1.000 | 3366 | tags=45%, list=28%, signal=62% |
| 53 | KEGG\_PATHWAYS\_IN\_CANCER |  | 249 | 0.25 | 1.09 | 0.261 | 0.675 | 1.000 | 2375 | tags=24%, list=20%, signal=29% |
| 54 | KEGG\_GLYCEROPHOSPHOLIPID\_METABOLISM |  | 58 | 0.30 | 1.07 | 0.334 | 0.711 | 1.000 | 1925 | tags=29%, list=16%, signal=35% |
| 55 | KEGG\_SMALL\_CELL\_LUNG\_CANCER |  | 71 | 0.29 | 1.06 | 0.351 | 0.719 | 1.000 | 1587 | tags=18%, list=13%, signal=21% |
| 56 | KEGG\_TYPE\_II\_DIABETES\_MELLITUS |  | 36 | 0.33 | 1.06 | 0.367 | 0.708 | 1.000 | 2810 | tags=36%, list=23%, signal=47% |
| 57 | KEGG\_LEISHMANIA\_INFECTION |  | 40 | 0.32 | 1.06 | 0.370 | 0.715 | 1.000 | 1216 | tags=18%, list=10%, signal=19% |
| 58 | KEGG\_PROTEIN\_EXPORT |  | 21 | 0.36 | 1.05 | 0.380 | 0.724 | 1.000 | 5014 | tags=71%, list=42%, signal=122% |
| 59 | KEGG\_BUTANOATE\_METABOLISM |  | 21 | 0.37 | 1.04 | 0.403 | 0.732 | 1.000 | 165 | tags=10%, list=1%, signal=10% |
| 60 | KEGG\_OOCYTE\_MEIOSIS |  | 82 | 0.27 | 1.03 | 0.391 | 0.738 | 1.000 | 4698 | tags=49%, list=39%, signal=80% |
| 61 | KEGG\_INSULIN\_SIGNALING\_PATHWAY |  | 107 | 0.25 | 1.01 | 0.426 | 0.797 | 1.000 | 2430 | tags=23%, list=20%, signal=29% |
| 62 | KEGG\_LONG\_TERM\_POTENTIATION |  | 47 | 0.29 | 1.00 | 0.462 | 0.811 | 1.000 | 2368 | tags=21%, list=20%, signal=26% |
| 63 | KEGG\_COMPLEMENT\_AND\_COAGULATION\_CASCADES |  | 40 | 0.30 | 0.99 | 0.473 | 0.822 | 1.000 | 2157 | tags=30%, list=18%, signal=36% |
| 64 | KEGG\_MAPK\_SIGNALING\_PATHWAY |  | 179 | 0.23 | 0.99 | 0.487 | 0.813 | 1.000 | 2466 | tags=22%, list=21%, signal=28% |
| 65 | KEGG\_INOSITOL\_PHOSPHATE\_METABOLISM |  | 49 | 0.29 | 0.98 | 0.480 | 0.807 | 1.000 | 3144 | tags=39%, list=26%, signal=52% |
| 66 | KEGG\_TRYPTOPHAN\_METABOLISM |  | 26 | 0.32 | 0.97 | 0.500 | 0.831 | 1.000 | 1295 | tags=19%, list=11%, signal=22% |
| 67 | KEGG\_NON\_HOMOLOGOUS\_END\_JOINING |  | 11 | 0.40 | 0.97 | 0.495 | 0.828 | 1.000 | 3591 | tags=73%, list=30%, signal=104% |
| 68 | KEGG\_AXON\_GUIDANCE |  | 100 | 0.25 | 0.97 | 0.519 | 0.817 | 1.000 | 2715 | tags=31%, list=23%, signal=40% |
| 69 | KEGG\_GLYOXYLATE\_AND\_DICARBOXYLATE\_METABOLISM |  | 14 | 0.38 | 0.97 | 0.500 | 0.805 | 1.000 | 2152 | tags=36%, list=18%, signal=43% |
| 70 | KEGG\_CHEMOKINE\_SIGNALING\_PATHWAY |  | 119 | 0.24 | 0.96 | 0.539 | 0.816 | 1.000 | 2430 | tags=27%, list=20%, signal=33% |
| 71 | KEGG\_PYRIMIDINE\_METABOLISM |  | 80 | 0.25 | 0.96 | 0.537 | 0.809 | 1.000 | 904 | tags=9%, list=8%, signal=9% |
| 72 | KEGG\_O\_GLYCAN\_BIOSYNTHESIS |  | 22 | 0.33 | 0.95 | 0.532 | 0.825 | 1.000 | 3492 | tags=50%, list=29%, signal=70% |
| 73 | KEGG\_VIRAL\_MYOCARDITIS |  | 33 | 0.29 | 0.93 | 0.569 | 0.864 | 1.000 | 2422 | tags=27%, list=20%, signal=34% |
| 74 | KEGG\_ARRHYTHMOGENIC\_RIGHT\_VENTRICULAR\_CARDIOMYOPATHY\_ARVC |  | 52 | 0.27 | 0.92 | 0.586 | 0.857 | 1.000 | 2131 | tags=27%, list=18%, signal=33% |
| 75 | KEGG\_N\_GLYCAN\_BIOSYNTHESIS |  | 40 | 0.28 | 0.92 | 0.576 | 0.847 | 1.000 | 1624 | tags=15%, list=14%, signal=17% |
| 76 | KEGG\_CIRCADIAN\_RHYTHM\_MAMMAL |  | 12 | 0.37 | 0.92 | 0.572 | 0.845 | 1.000 | 1561 | tags=33%, list=13%, signal=38% |
| 77 | KEGG\_PHOSPHATIDYLINOSITOL\_SIGNALING\_SYSTEM |  | 65 | 0.25 | 0.91 | 0.618 | 0.853 | 1.000 | 1765 | tags=23%, list=15%, signal=27% |
| 78 | KEGG\_EPITHELIAL\_CELL\_SIGNALING\_IN\_HELICOBACTER\_PYLORI\_INFECTION |  | 59 | 0.25 | 0.90 | 0.638 | 0.868 | 1.000 | 1837 | tags=19%, list=15%, signal=22% |
| 79 | KEGG\_BLADDER\_CANCER |  | 32 | 0.28 | 0.87 | 0.654 | 0.927 | 1.000 | 4198 | tags=53%, list=35%, signal=81% |
| 80 | KEGG\_COLORECTAL\_CANCER |  | 51 | 0.25 | 0.87 | 0.690 | 0.930 | 1.000 | 1737 | tags=22%, list=14%, signal=25% |
| 81 | KEGG\_LINOLEIC\_ACID\_METABOLISM |  | 10 | 0.36 | 0.85 | 0.659 | 0.961 | 1.000 | 448 | tags=20%, list=4%, signal=21% |
| 82 | KEGG\_MTOR\_SIGNALING\_PATHWAY |  | 45 | 0.25 | 0.84 | 0.726 | 0.964 | 1.000 | 2017 | tags=20%, list=17%, signal=24% |
| 83 | KEGG\_LEUKOCYTE\_TRANSENDOTHELIAL\_MIGRATION |  | 81 | 0.22 | 0.84 | 0.776 | 0.959 | 1.000 | 2398 | tags=28%, list=20%, signal=35% |
| 84 | KEGG\_GLYCOSAMINOGLYCAN\_BIOSYNTHESIS\_HEPARAN\_SULFATE |  | 18 | 0.30 | 0.83 | 0.690 | 0.954 | 1.000 | 783 | tags=17%, list=7%, signal=18% |
| 85 | KEGG\_ALZHEIMERS\_DISEASE |  | 111 | 0.21 | 0.83 | 0.820 | 0.948 | 1.000 | 2366 | tags=17%, list=20%, signal=21% |
| 86 | KEGG\_NUCLEOTIDE\_EXCISION\_REPAIR |  | 38 | 0.25 | 0.83 | 0.749 | 0.946 | 1.000 | 3521 | tags=32%, list=29%, signal=45% |
| 87 | KEGG\_FC\_EPSILON\_RI\_SIGNALING\_PATHWAY |  | 56 | 0.23 | 0.82 | 0.796 | 0.951 | 1.000 | 2169 | tags=23%, list=18%, signal=28% |
| 88 | KEGG\_GAP\_JUNCTION |  | 63 | 0.22 | 0.82 | 0.799 | 0.945 | 1.000 | 2262 | tags=21%, list=19%, signal=25% |
| 89 | KEGG\_OTHER\_GLYCAN\_DEGRADATION |  | 12 | 0.33 | 0.81 | 0.712 | 0.949 | 1.000 | 130 | tags=8%, list=1%, signal=8% |
| 90 | KEGG\_VASOPRESSIN\_REGULATED\_WATER\_REABSORPTION |  | 37 | 0.24 | 0.80 | 0.789 | 0.958 | 1.000 | 2385 | tags=27%, list=20%, signal=34% |
| 91 | KEGG\_ANTIGEN\_PROCESSING\_AND\_PRESENTATION |  | 26 | 0.26 | 0.79 | 0.777 | 0.966 | 1.000 | 2348 | tags=27%, list=20%, signal=33% |
| 92 | KEGG\_TASTE\_TRANSDUCTION |  | 17 | 0.29 | 0.79 | 0.766 | 0.955 | 1.000 | 2778 | tags=41%, list=23%, signal=53% |
| 93 | KEGG\_MATURITY\_ONSET\_DIABETES\_OF\_THE\_YOUNG |  | 15 | 0.30 | 0.78 | 0.755 | 0.956 | 1.000 | 2030 | tags=33%, list=17%, signal=40% |
| 94 | KEGG\_SELENOAMINO\_ACID\_METABOLISM |  | 19 | 0.28 | 0.78 | 0.770 | 0.955 | 1.000 | 972 | tags=16%, list=8%, signal=17% |
| 95 | KEGG\_PROTEASOME |  | 31 | 0.25 | 0.77 | 0.815 | 0.950 | 1.000 | 9072 | tags=100%, list=76%, signal=408% |
| 96 | KEGG\_VEGF\_SIGNALING\_PATHWAY |  | 55 | 0.22 | 0.77 | 0.860 | 0.941 | 1.000 | 2764 | tags=25%, list=23%, signal=33% |
| 97 | KEGG\_UBIQUITIN\_MEDIATED\_PROTEOLYSIS |  | 111 | 0.19 | 0.74 | 0.948 | 0.973 | 1.000 | 1692 | tags=12%, list=14%, signal=14% |
| 98 | KEGG\_PROGESTERONE\_MEDIATED\_OOCYTE\_MATURATION |  | 67 | 0.20 | 0.74 | 0.914 | 0.969 | 1.000 | 3700 | tags=36%, list=31%, signal=51% |
| 99 | KEGG\_RENAL\_CELL\_CARCINOMA |  | 62 | 0.20 | 0.72 | 0.922 | 0.975 | 1.000 | 2430 | tags=23%, list=20%, signal=28% |
| 100 | KEGG\_PROSTATE\_CANCER |  | 75 | 0.19 | 0.72 | 0.935 | 0.967 | 1.000 | 2759 | tags=23%, list=23%, signal=29% |
| 101 | KEGG\_MELANOGENESIS |  | 63 | 0.20 | 0.72 | 0.931 | 0.966 | 1.000 | 1397 | tags=13%, list=12%, signal=14% |
| 102 | KEGG\_SNARE\_INTERACTIONS\_IN\_VESICULAR\_TRANSPORT |  | 30 | 0.23 | 0.71 | 0.894 | 0.958 | 1.000 | 3444 | tags=37%, list=29%, signal=51% |
| 103 | KEGG\_GLYCOSPHINGOLIPID\_BIOSYNTHESIS\_GANGLIO\_SERIES |  | 13 | 0.27 | 0.69 | 0.874 | 0.975 | 1.000 | 153 | tags=8%, list=1%, signal=8% |
| 104 | KEGG\_BIOSYNTHESIS\_OF\_UNSATURATED\_FATTY\_ACIDS |  | 19 | 0.22 | 0.62 | 0.947 | 1.000 | 1.000 | 2960 | tags=26%, list=25%, signal=35% |
| 105 | KEGG\_GLYCINE\_SERINE\_AND\_THREONINE\_METABOLISM |  | 19 | 0.22 | 0.62 | 0.945 | 1.000 | 1.000 | 1941 | tags=21%, list=16%, signal=25% |
| 106 | KEGG\_THYROID\_CANCER |  | 24 | 0.21 | 0.61 | 0.961 | 0.993 | 1.000 | 2683 | tags=25%, list=22%, signal=32% |
| 107 | KEGG\_RNA\_DEGRADATION |  | 49 | 0.17 | 0.59 | 0.985 | 0.994 | 1.000 | 4246 | tags=39%, list=35%, signal=60% |
| 108 | KEGG\_REGULATION\_OF\_AUTOPHAGY |  | 17 | 0.21 | 0.58 | 0.959 | 0.988 | 1.000 | 1419 | tags=12%, list=12%, signal=13% |
| 109 | KEGG\_HEDGEHOG\_SIGNALING\_PATHWAY |  | 33 | 0.15 | 0.48 | 0.997 | 0.996 | 1.000 | 4561 | tags=48%, list=38%, signal=78% |
Table: Gene sets enriched in phenotype **1 (5 samples) **[plain text format]****
